# Caring Behavior Coding Scheme based on Swanson’s Theory of Caring – development and testing among undergraduate nursing students

**DOI:** 10.1111/scs.12927

**Published:** 2020-10-30

**Authors:** Sophie Mårtensson, Eric A. Hodges, Susanne Knutsson, Carina Hjelm, Anders Broström, Kristen M. Swanson, Maria Björk

**Affiliations:** ^1^ Department of Nursing Science School of Health and Welfare Jönköping University Jönköping Sweden; ^2^ CHILD Research Group Jönköping University Jönköping Sweden; ^3^ School of Nursing The University of North Carolina at Chapel Hill Chapel Hill NC USA; ^4^ Department of Health and Caring Sciences Faculty of Health and Life Sciences Linnaeus University Växjö Sweden; ^5^ Department of Health, Medicine and Caring Sciences Linköping University Linköping Sweden; ^6^ Department of Clinical Neurophysiology Linköping University Hospital Linköping Sweden; ^7^ Seattle University Seattle WA USA

**Keywords:** behavioural coding, caring behaviour, observational methods, Swanson’s Theory of Caring, simulation, healthcare providers, undergraduate nursing student

## Abstract

**Rationale:**

To maintain patients’ dignity and well‐being and alleviate suffering, it is essential that healthcare providers engage in caring behaviours. Yet, every year patient boards receive an increasing number of complaints from patients and significant others regarding healthcare providers’ non‐caring behaviours. Defining and measuring both verbal and nonverbal caring and non‐caring behaviour in healthcare delivery is vital to address such complaints. However, no studies were found that incorporated a comprehensive theory of caring to code encounters between healthcare providers and patients.

**Aim:**

The aim was to develop and test a Caring Behavior Coding Scheme based on Swanson’s Theory of Caring.

**Method:**

An instrument development process was used for behavioural coding including observational data from thirty‐eight video recordings collected in an undergraduate nursing course at a Swedish University. The observational data involved interactions between undergraduate nursing students and a standardised patient.

**Result:**

The Caring Behavior Coding Scheme (the CBCS), contains seventeen verbal and eight nonverbal behavioural codes, categorised as caring and non‐caring in accordance with Swanson’s Theory of Caring. Content and face validity were assessed. Timed‐event sequential continuous coding was performed in INTERACT software. The coder achieved excellent agreement with the developed gold standard (*k* = 0.87) and excellent mean inter‐rater reliability (*k* = 0.82). All domains in Swanson’s Theory of Caring were observed and coded in the interaction.

**Discussion/Conclusion:**

The CBCS is a theory‐based instrument that contributes to research on healthcare providers’ behavioural encounters. It uses verbal and nonverbal caring and non‐caring behavioural codes to assess the alignment of both the theory and practice of caring. The CBCS can contribute to both development and measurement of interventions focused on improving healthcare providers’ caring behaviour with the intended outcome of patient well‐being.

## Introduction

The behaviours of healthcare providers affect how patients participate in and experience care situations. The manner in which a healthcare provider interacts with care recipients can either increase or decrease patients’ vulnerability and suffering (1, 2). When patients experience being seen, heard and trusted by healthcare providers, they are more likely to follow treatment strategies; conversely, patients who experience the opposite are more reluctant to seek care, which can lead to delayed diagnoses and treatments (3). During the last few decades, the Swedish healthcare system has gone through organisational changes and become more dynamic and complex (4) with fewer hospital beds, shorter lengths of stay, and a shift towards home‐based care (5). With these changes in care delivery, the quality of care is dependent on the initiatives of individuals (e.g. healthcare providers and patients) rather than the organisational level (6). Patients’ experiences of disrespect, a lack of professional competence and poor organisational commitment to quality improvement work are associated with increased numbers of adverse patient outcomes and patient complaints (4). The same phenomena have also been reported in other high‐income countries with an increasing amount of complaints to the patient boards, which receive complaints from patients and significant others regarding healthcare providers’ non‐caring behaviour and lack of professional competence (2, 7). These complaints demonstrate a need for strategies to increase and enhance healthcare providers’ awareness of their own verbal and nonverbal caring behaviours in order to maintain patients’ dignity and well‐being and alleviate suffering (2, 3).

In the discipline of nursing, theoretical structures of caring have been established as the central core concept of guidance in all nurses’ work (8, 9). Watson (10) described in her Grand Theory of Human Caring that the human being is a spiritual wholeness that cannot be divided into body and soul. Informed by Watson, Swanson empirically developed a middle range Theory of Caring (11), based on three phenomenological studies in separate perinatal contexts (12, 13). Swanson’s Theory of Caring demonstrates a lower level of abstraction than Watson’s Grand Theory of Human Caring and highlights specific phenomena and examples of what it means for nurses to practice caring. The theory defines caring as a ‘nurturing way of relating to a valued other towards whom one feels a personal sense of commitment and responsibility’ (13, p. 165) and is described in five conceptual domains (11) (Table 1).

**Table 1:** Description of domains and subdomains in Swanson’s Theory of Caring (12, 13)

Domain	Subdomain
*Maintaining belief* Sustaining faith in the other’s capacity to get through an event or transition and face a future with meaning	Believing in or holding in esteem Offering a hope‐filled attitude Offering realistic optimism Helping find meaning Going the Distance
*Knowing* Striving to understand an event as it has meaning in the life of the other’s	Avoiding assumption Centring on the one cared for Assessing needs Seeking cues Engaging self and other
*Being* *with* Being emotionally present to the other’s	Being there Conveying availability Sharing feelings Not burdening Enduring with
*Doing* *for* Doing for the other’s as he/she would do for themselves	Comforting Anticipating Performing competently/skillfully Protecting Preserving dignity
*Enabling* Facilitating the other’s passage through life transitions and unfamiliar events	Informing/Explaining Supporting/Allowing Focusing Generating alternatives/Thinking it through Validating giving feedback

The role of and needs for caring have been discussed through the years (14). Kuhn (15) declared in his seminal work that there must be an underlying theory‐based core concept in how to interpret and understand behaviour. Watson (16) stated when the knowledge from caring theories is implemented in practice, there are differences in the quality of caring behaviour. Still there exists disagreement within and outside of nursing on the role of caring behaviour in personal and professional encounters. Some refer to nursing as a practical profession alone that is composed of clinical tasks without a need for theoretical knowledge (14). However, patients have reported that they need not only clinically competence nurses, but nurses who provide caring interactions with compassion (13). This reveals the need to develop a theory‐based instrument which can capture and measure the complex phenomena of caring behaviour (14, 16), as well as capture and describe behaviour patterns which are universal expressions of caring, since caring behaviour is not unique to nursing (12).

Existing behavioural coding schemes for use in analysing healthcare providers’ verbal and nonverbal behaviour are primarily based on communication theories and interviewing techniques in the medical context (17, 18). The Roter Interaction analysis system (RIAS) is the mostly cited coding scheme in medical literature; it codes physician–patient dialogue in medical care (19). The Medical Interaction Process System (MIPS) codes communication skills between physicians and patients with cancer (20). The Four Habits Model (4HCS) codes physicians’ behaviours and skills associated with patient‐centred care (21). All these behavioural coding schemes aim to describe and analyse a variety of communication skills specific to healthcare providers’ behaviour (e.g. questioning and listening). However, they do not depict the qualitative dimensions of caring behaviour in communication (e.g. competence and compassion).

There are instruments developed around caring behaviours in nursing (16). For example, the caring behaviours instrument is an observer rating instrument that evaluates verbal and nonverbal caring and non‐caring behaviour demonstrated by the nurse in patient care (22). However, the caring behaviours instrument is not based on a specific caring theory that captures fully the theoretical dimensions of caring phenomena. Dunnington et al. (23) revised the caring behaviours instrument by adding four of the ten dimensions in Watson’s Grand Theory of Human Caring (24, 25). Watson (16) stated it is essential to develop measurements based on caring theory in order to understand healthcare providers’ encounters with patients. Swanson’s Theory of Caring is a middle range theory that reflects all ten dimensions of Watson’s Grand Theory of Human Caring of caring at a lower level of abstraction (11) and has broad application across different healthcare settings and healthcare providers in several countries around the globe (26). Bai et al. (27) developed a behavioural coding scheme based on Swanson’s Theory of Caring to measure the responsiveness between parent and child during painful cancer treatments. However, no studies were found describing a behavioural coding scheme, based on Swanson’s middle range theory and focusing on encounters between healthcare providers and patients.

## Aim

The aim was to develop and test a caring behaviour coding scheme based on Swanson’s Theory of Caring.

## Method

### Design and sample

This study used an instrument development design (28) and followed the process for developing a behavioural coding scheme as outlined by Chorney et al. (29). In the development process, observational data from thirty‐eight existing video recordings collected in a 7.5 credit course in semester four of undergraduate nursing education at a Swedish University were used. In Sweden, undergraduate nursing education consists of six semesters (180 credits) and leads to a professional nursing degree and a Bachelor of Science (30). When attending the 7.5 credit course focusing on caring behaviour, the undergraduate nursing student had completed 45 credits in the main area of nursing (of total 120 credits), 30 credits in medical science and 15 credits in social behaviour science (of total 60 credits). In total, 20 undergraduate nursing students attended the course. Of these, 19 students, four men and 15 women (ages 21–31 years), agreed to participate in the study.

### Ethical approval

This study followed the Helsinki declaration (30). All students in the course received oral and written information about the study which emphasised confidentiality that participation was voluntary and that non‐participation or withdrawal would not affect their grades. After having had opportunity to ask questions, students gave their written informed consent. The study was approved by the Research Ethics Committee in Linköping, Sweden, (DNR 2017/503‐31).

### Observational setting

At the beginning and the end of the course, undergraduate nursing students interacted with a standardised patient in a caring behaviour simulation. The scenario and acting script for the standardised patient were written and authenticated by three experienced registered nurses and academics (i.e. registered nurse teachers), who had extensive pedagogical experience from education in clinical and simulation situations. The scenario depicts a 70‐year‐old woman who was recently discharged after undergoing planned hip surgery without complications. She lives alone in a house in the countryside and receives visits from the home service. During one visit, she was not feeling well, as she felt anxious, dizzy and nauseated. Based on this, the student was asked to visit her. The simulation scenario lasted for 8 minutes and was video recorded using three GoPro Hero 5 Session (US) cameras placed at different points of view around the room.

### Instrument development process

The instrument development process included four steps as outlined by Chorney et al. (29). *Step one,* refining the research question, involves determining what behaviours of interest to code, who, when, and how to observe, and considering the analytic plan. *Step two*, developing the coding manual, involves developing a list of operational definitions for each code, sampling strategy, and providing instructions on implementations of the coding scheme. *Step three*, piloting and refining the coding manual, involves application of the coding scheme to a sample of observations with resource constraints considered. Lastly, *step four,* implementing the coding scheme, involves defining the coder requirements, training the coder, coding the data, and checking agreement, and examining validity, analysing and reporting data. In steps three and four, INTERACT (Mangold International, Germany) software was used. This allowed the researcher (first author) to perform synchronised viewing and analysis of video recordings and audio files, content coding and event logging, which generates qualitative and quantitative results for use in observational research (31).

## Results

### The development process of the Caring Behavior Coding Scheme

#### Step one: Refining the research question

Discussions were held between the authors of how to best capture the caring phenomena in healthcare providers’ caring‐ and non‐caring behaviour through Swanson’s Theory of Caring (12, 13). Verbal and nonverbal caring and non‐caring behavioural nominal codes (i.e. codes describing the behaviour to be scored) (29) were developed to categorise healthcare providers’ encounters in accordance with the domains defined in Swanson’s Theory of Caring (12, 13). A suitable interaction period that allows verbal and nonverbal expression to be described is about 5 minutes (32). In the application to video‐recorded observational data, the coding started when the undergraduate nursing student entered the simulation area and stopped after 6 minutes. An analytic plan was considered which contained who should code and a preliminary timeline for coding video‐recorded observational data.

#### Step two: Developing the coding manual

Bakeman et al. (33) have established two forms of behavioural codes: physically based codes vs. socially based codes and granularity micro vs. macro. Physically based codes apply to explicit physical actions (e.g. the healthcare provider stands up one metre in front of the patient); conversely, socially based codes apply to behaviours at a broader level (e.g. the healthcare provider stands up close enough to touch the patient). Micro codes capture behaviours at their most specific level (e.g. utterance to utterance) whereas macro codes capture behaviours on a more general level (e.g. healthcare provider has a friendly facial expression) (33). Given the theoretical dimensions of caring behaviour in Swanson’s Theory of Caring (12, 13), only macro socially based behavioural codes were developed.

To each domain’s (e.g. knowing) subdomains (e.g. avoiding assumption) (Table 1), operational definitions were developed focusing on verbal and nonverbal caring and non‐caring behaviour. This was accomplished by reviewing previous studies guided by Swanson’s Theory of Caring. Concurrently, for behavioural code generation, movies, art and literature depicting healthcare providers’ verbal and nonverbal caring and non‐caring behaviours were reviewed. The generated verbal and nonverbal caring and non‐caring behavioural codes were assembled and structured into relevant subdomains in Swanson’s Theory of Caring (12, 13). This process involved discussions between the authors and Dr. Swanson to deepen and confirm the developed operational definitions and the generated behavioural codes in their relation to each subdomain in Swanson’s Theory of Caring (12, 13). Additionally, all verbal and nonverbal caring and non‐caring behavioural codes were ensured to be mutually exclusive that is, each behaviour has its own code and the codes are exhaustive for all behaviours of interest (33).

As the coding schema evolved, a code manual was written which contains detailed descriptions of how to apply and use the Caring Behavior Coding Scheme (the CBCS) to observational data. The sampling strategy involved timed‐event sequential continuous coding, which records any occurrence of a behavioural code in the stream of behaviour and provides data on frequency/event, duration/state and order of behaviour (29). In the CBCS, all verbal caring and non‐caring behavioural codes are event (frequency) codes and all nonverbal caring and non‐caring behavioural codes are state (duration) codes.

#### Step three: Piloting and refining the coding manual

**Figure 1:**
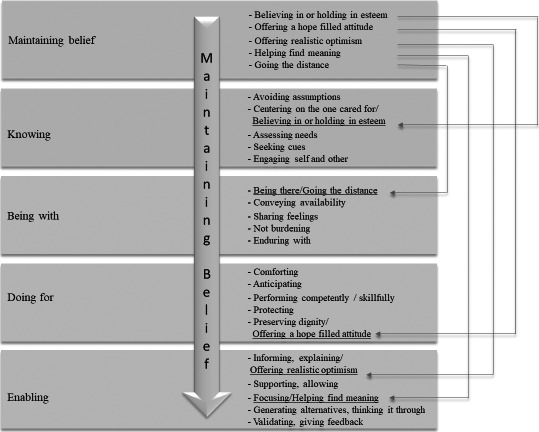
Description of how the domain Maintaining Belief with related subdomains is intertwined in the other domains with related subdomains in the CBCS.

During the pilot and refinement of the coding manual, the first author applied the CBCS to nine randomly selected video recordings out of the subsample of 38 video recordings using INTERACT software. In this process, it became clear that the developed behavioural codes could be categorised under more than one domain with related subdomains. Each subdomain, its operational definition and behavioural codes were reviewed during coding and in discussions between the authors to refine the operational definitions and behavioural codes. This iterative process of back and forth of coding and adjustments led to the decision to intertwine the domain, *Maintaining belief,* with related subdomains into the other domains with related subdomains (Fig. 1). This decision was discussed with Dr. Swanson who agreed and noted that Maintaining Belief is an internally experienced perception on the part of the healthcare provider that is expressed by knowing, being with, doing for and enabling the recipient of care.

The refined CBCS contains twenty‐five behavioural codes: seventeen verbal caring and non‐caring behavioural codes and eight nonverbal caring and non‐caring behavioural codes (Tables 2 and 3).

**Table 2:** Examples of verbal caring‐ and non‐caring behavioural codes in the CBCS

Code/Domain	Operational definition of domain	Code/Subdomain	Operational definition of subdomain	Example of behavioural codes ‐Caring behaviour	Example of behavioural codes ‐Non‐Caring behaviour
Knowing	Striving to understand an event as it has meaning in the life of the other’s	Centring on the one cared for/Believing in or holding in esteem	The verbalisation demonstrates respect, empathy, and compassion and genuinely tries to understand the person’s perspective. Focusing on the person and not themselves (Knowing). Views the person as a whole person regardless of condition or illness (Maintaining belief).	Listen to – let the person explain their situation and allows for complete response For example: ‘Okay’ ‘When I hear you say…’	Interrupts and/or changing topics when the person is explaining their situation and thereby misses opportunities to follow up what unfolds in the conversation For example: Interrupt Changing topics Miss opportunities ‘I feel dizzy, ohh you feel pain in your right leg’
Being with	Being emotionally present for the person	Sharing feelings	The verbalisation demonstrates in a warmth, compassionate way that they have a willingness to share feelings in an understanding way with the person. The rater/coder/viewer has a sense of mutuality and reciprocity when observing the interaction	Encourage expressions of feelings (such as joy, sadness, and worry) or share a lived experience (if that is appropriate) For example: Laughing together Crying together ‘I’m sorry to hear that you feel that way!’ ‘I’m sorry you have experienced that!’ ‘I have also…’ I know someone who…’	Belittling the person feelings For example: ‘That is nothing to worry about!’ ‘No in reality this is what happened…’ ‘Everything will be just fine!’ ‘My experience of this is that it is not that bad…’ ‘I think that you really should do this…’

**Table 3:** Examples of nonverbal caring and non‐caring behavioural codes in the CBCS

Code/Domain	Operational definition of domain	Code/Subdomain	Operational definition of subdomain	Example of behavioural codes ‐ Caring behaviour	Example of behavioural codes ‐ Non‐Caring behaviour
Knowing	Striving to understand an event as it has meaning in the life of the person	Seeking Cues	Demonstrates a genuine and warm attempt to sense and recognise the person’s nonverbal cues reflecting the person’s expression of concern, needs and/or stress	In a genuine and warm way, be aware of underlying cues Gazing at with warmth and friendliness	Does not show any concerns or tries to identify underlaying cues Gazing with harsh and no friendliness Flacking around/nervous impression Staring
Being with	Being emotionally present for the person	Being there/ Going the distance	Demonstrates in a warm, compassionate, and genuine way that they are being physically and mindfully present in this moment (Being with). Reflect their commitment to care for the person as best they can for the duration of the person’s treatment, no matter what happens (Maintaining belief).	Body posture and facial expression demonstrate a willingness, braveness and courage to be with the person Body posture is open and friendly Body posture is directed towards the person Facial expression demonstrates warmth and compassion	Demonstrates no willingness to be with the person and physically leaves the person alone when he/she needs the caring most Body posture is closed Body posture is directed away from the person Facial expression is harsh and not friendly

#### Step four: Implementing the coding scheme

Before applying the CBCS, content validity (28) was examined by Dr. Swanson, who confirmed that the CBCS captures the content of Swanson’s Theory of Caring (12, 13). Face validity (28) was conducted with two nurses uninvolved in the study whose work experiences ranged from 10 to 15 years in various healthcare settings (e.g. intensive acute care‐unit, and municipal healthcare). They confirmed that the CBCS coding scheme adequately captured caring behaviours and that the codes differentiated caring and non‐caring interactions.

The first author was trained in INTERACT software by reading manuals and had email conversation and video conferences with their technical support. The first author trained the coder, who has a PhD in nursing and who was not involved in the development of the CBCS nor the collection of observational data. During the training period, the first author and the coder communicated regularly through physical meetings and video conferences. The meetings contained an orientation to Swanson’s Theory of Caring (12, 13), the CBCS and INTERACT software. During the training period, the coder applied the CBCS to the same nine video recordings that were used during the development and refining process of the CBCS. Coding results were compared and discussed between coders. A gold standard video randomly extracted out of the 38 video recordings was developed by the first and last author using a consensus process when coding it. When the training period neared the end, the coder used the CBCS to code the gold standard video. The coder was considered trained at an adequate level when the level of agreement achieved a Cohen’s kappa at or above 0.80 (29, 33). The coder achieved Cohen’s kappa 0.87 in agreement with the gold standard and was considered sufficiently credentialed to conduct coding of fourteen randomly selected video observations out of a subsample of the remaining twenty‐eight video recordings in Interact software.

To estimate the inter‐rater reliability (IRR) between the coder and first author, Cohen’s kappa was analysed in INTERACT software for seven randomly selected video observations out of the fourteen coded video recordings (e.g. 50%). The IRR observer agreement of the seven randomly selected video observations had a mean value of *k* = 0.82 (range: 0.77–0.88). In the literature, a random sampling for IRR observer agreement is to be ten to thirty per cent of observational data and is at an adequate level when the measure value of Cohen’s kappa is above 0.80 (29, 33).

#### Testing the CBCS

Observational data from fourteen analysed video recordings were used in testing of the CBCS (Table 4). Observational data were analysed in INTERACT software for verbal (e.g. frequency) and nonverbal (e.g. duration) caring and non‐caring behaviours in accordance with the domains defined in Swanson’s Theory of Caring (12, 13). Testing the CBCS displayed a slight majority of verbal behavioural codes were caring (54%) as opposed to non‐caring (46%). One hundred per cent of nonverbal behavioural codes were caring.

**Table 4:** Present the testing of CBCS on fourteen analysed video recordings

Domain	Subdomain	Caring behaviour		Non‐caring behaviour	
		Verbal^a^ (N = 217) (%)	Nonverbal^b^(%)	Verbal^a^ (N = 117) (%)	Nonverbal^b^ (%)
Knowing	Avoiding assumptions	40 (18)		3 (3)	
	Centring on the one being cared for/Believing in or holding in esteem	50 (24)	19	41 (35)	
	Assessing needs	25 (11)		19 (16)	
	Seeking cues		15	1 (1)	
	Engaging the self and Other		19		
Being with	Being there/Going the distance		23		
	Conveying availability	16 (7)	1	1 (1)	
	Sharing feelings	14 (6)	23	19 (16)	
	Not burdening	1 (1)			
	Enduring with				
Doing for	Comforting	19 (9)		9 (7)	
	Anticipating				
	Performing competently/skillfully	23 (10)		9 (7)	
	Protecting	2 (1)		3 (2)	
	Preserving dignity/Offering a hope‐filled attitude	5 (2)			
Enabling	Informing/explaining/Offering realistic optimism	20 (9)			
	Supporting allowing				
	Focusing/Helping find meaning	3 (2)		13 (11)	
	Generating alternatives/thinking it through				
	Validating/giving feedback				

^a^ Frequency.

^b^ Duration

In the domain *Knowing,* all subdomains were represented. The most frequently occurring verbal behavioural code was displayed in the intertwined subdomain *centring on the one being cared for/believing in or holding in esteem*. The second most occurring behavioural code was in the subdomain, *assessing needs*. In the domain *Being with,* the most frequently occurring behavioural codes were displayed in the subdomain *sharing feelings* and no codes were displayed in the subdomain *enduring with*. In the domain *Doing for,* the most frequently occurring behavioural code was in the subdomain *performing competently/skillfully* and no codes were displayed in the subdomain *anticipating*. In the domain *Enabling,* codes were displayed in the intertwined subdomains *informing/explaining/offering realistic optimism* and *focusing/helping find meaning,* and in three subdomains, there were no codes displayed. The most time spent in nonverbal behavioural codes occurred in caring behaviour in the domain *Being with* in two subdomains the intertwined subdomains *being there/going the distance* and *sharing feelings*.

## Discussion

The present study describes the development and testing of the CBCS based on Swanson’s Theory of Caring (12, 13). Existing video‐recorded observational data collected during undergraduate nursing education at a Swedish University were analysed using the sampling strategy of timed‐event sequential continuous coding in INTERACT software. The CBCS was developed to uncover and explore the theoretical dimension of caring phenomena with concrete behavioural code descriptions for verbal and nonverbal caring and non‐caring behaviour in healthcare providers’ encounters with patients in accordance with the domains defined in Swanson’s Theory of Caring (12, 13). There is consensus in the literature that healthcare providers should encounter patients with caring behaviour (14). Yet, surprisingly, there is little guidance of how healthcare providers can use and apply caring behaviour (34). Watson (17) stated that it is essential to develop measurements based on caring theory in order to understand healthcare providers’ encounters with patients. Swanson’s Theory of Caring served as the theoretical foundation to the CBCS by being a middle range theory of caring with high descriptive value in caring phenomena and a broad range of application (26, 35). Additionally, the development of the CBCS followed the description steps outlined by Chorney et al. (29, 33). The result provides an example for instrument development process of a behavioural coding scheme based on a middle range theory of caring.

Itemized in Swanson’s Theory of Caring (12, 13), caring behaviour occurs within the encounter and leads to an intended outcome of patient well‐being (13). In previous literature, the five caring domains in Swanson’s Theory of Caring (12, 13) were presented separately; however, they are described as intertwined and therefore not mutually exclusive (26, 35). Swanson (13) stated that in the domain *Maintaining belief,* it is not the goal to give the other’s life meaning; instead, it is to strive to know, be with, do for, and enable the other to attain, maintain or regain meaning in their experiences of health and illness. Consequently, in the CBCS the domain *Maintaining belief* with its related subdomains is intertwined with the other domains and their related subdomains (Fig. 1). This intertwining of the domain *Maintaining belief* with its related subdomains enables the CBCS to depict the wholeness and core essence in caring.

The CBCS contain seventeen verbal and eight nonverbal caring and non‐caring macro socially‐based behavioural codes. Why no micro physically based behavioural codes were developed in the CBCS is due to the theoretical dimensions of caring described in the caring literature. In comparison with existing coding schemes, RIAS contains twenty‐eight verbal behavioural codes (19) and the 4HCS contains 23 verbal and nonverbal behavioural codes (21). The CBCS consists of a suitable number of items and contributes to healthcare providers’ behavioural encounter studies by adding 25 verbal and nonverbal macro socially based behavioural codes in accordance with Swanson’s Theory of Caring.

Content validity for the CBCS involved continuous discussions during the development phase with Dr. Swanson, who confirmed that the codes in the CBCS captured the domains described in her Theory of Caring (12, 13). Even though face validity is not considered as strong evidence for an instrument’s validity (28), the authors considered it important to evaluate face validity with two clinical nurses to ensure the CBCS captured relevant caring behaviours in clinical practice, which they confirmed. The CBCS showed acceptable reliability. The coder was considered fully trained by the first author the gold standard video observation was coded with the agreement *k* = 0.87. Considering the fact that it is difficult to become a reliable coder (29) the achieved Cohen’s kappa value is excellent. To evaluate if the CBCS is a reliable instrument and that the coder does not drift, usually between 10 and 25% of all observational data are coded for observer agreement (29). In the present study, fifty per cent of the analysed observational data were randomly assessed due to the small amount of observational data being coded, where the mean inter‐rater reliability had excellent agreement with *k* = 0.82. Taken together, these findings provide support that the CBCS is a valid and reliable theory‐derived instrument that provides useful data when evaluating healthcare providers’ verbal and nonverbal caring and non‐caring behaviour. The validity and reliability of the CBCS support its use in observational behavioural research.

The testing results of the CBCS are promising. All domains in Swanson’s Theory of Caring (12, 13) were represented and only five subdomains were not represented (Table 4). This may be due to the context of the observational setting and the observational sample. Furthermore, no nonverbal non‐caring behaviour was displayed; this may be different in other observational settings context. The most frequent behavioural code displayed was in the domain *Knowing*. Swanson (12) stated that when *Knowing* occurs, it is with the desire to understand the personal reality of the one receiving care. The most frequently used behavioural code was the intertwined subdomain *centring on the one being cared for/believing in or holding in esteem*. Swanson (13) stated for a respectful and trustworthy relationship to occur in encounters, the healthcare provider needs to be a present compassionate non‐judgmental listener for the one receiving care to genuinely feel accepted for who they are as a person. The rarest behavioural codes were displayed in the domain *Enabling*. Swanson (12) stated that an enabling provider is one who ‘downloads’ his or her expert knowledge to the one receiving care with the intention of enhancing the capacity of the other to participate in their own care. The observational sample and observational setting in this study may not have been of a nature to reflect the theoretical dimension of caring phenomena defined in the domain *Enabling*. The testing results support the CBCS to be a theory‐based instrument that assesses and captures the process of caring in accordance with Swanson’s Theory of Caring (12, 13).

Almost half a century ago, Leininger (36) stated that caring behaviour should be based on knowledge gained during undergraduate nursing education. Throughout their education, undergraduate nursing students are exposed to numerous caring theories. Caring has been established as the central core concept of guidance in all nurses’ work yet nurses often lack a deeper understanding of caring phenomena due to the many interpretation of caring concepts in nursing literature (37). Clark (38) argued that undergraduate nursing students are not taught how to use caring behaviour when encountering patients. Benner (39) stated that the nurse passes through five development phases, which are the novice, advanced beginner, competent, proficient and expert. The novice nurse has no or little experience in a situation and the expert nurse has deep knowledge and understanding of the total situation. Combining the theoretical dimension of caring phenomena with concrete caring behaviour is described as necessary for the undergraduate nursing students’ development (14, 40). The use of the CBCS in caring behaviour simulation provides important insights through coding and quantification of verbal and nonverbal caring and non‐caring behaviour in the novice undergraduate nursing student. The results of the present study paired with the increasing amount of complaints received annually by patient boards from patient and significant others regarding healthcare non‐caring behaviour and lack of professional competence (2, 7) emphasises the importance of why caring behaviour needs to be a focal area in healthcare providers’ education (37). The results from this study demonstrate a way of capturing caring behaviour as an importance aspect of healthcare delivery. We hope this study will inspire future research into how caring behaviour can best be taught. Caring applies to all healthcare providers since caring behaviour is not unique to nursing (12).

### Limitations

This study has several limitations. One challenge was the language barrier when interpreting words and the underlying meaning of the wholeness and core meaning in Swanson’s Theory of Caring (12, 13). Therefore, ongoing discussions during the instrument development process of the CBCS were held with the second author, a native language speaker and theory developer Dr. Swanson in order to deepen and confirm the interpretation in Swanson Theory of Caring (12, 13). Moreover, all authors are nurses with various experiences within healthcare settings and education. Thus, the terminology used in the CBCS represents terminology denoting nursing knowledge. A broader interpretation of the CBCS may have been enhanced by inviting people with diverse expertise outside nursing to discuss the interpretation of the CBCS.

Developing a behavioural coding scheme based on macro socially based codes can be considered a limitation. Often socially based behavioural codes need more human judgment from the coder than physically based behavioural codes. Furthermore, the coder also needs to have professional knowledge in the area to apply the socially based behavioural codes with reliability (29). Accordingly, the trained coder has a PhD in nursing and extensive knowledge and experiences of simulation scenario in educational settings. Moreover, the validation of the CBCS needs to be further strengthened through construct validity analysis in comparison with other caring behavioural codes, for example the Caring Professional Scale (CPS) (16) or Caring Behaviors Inventory (CBI) (41). Finally, the small amount of analysed video recordings and our observational sample may raise questions of whether the CBCS can be generalised to other populations and contexts. Future work should include a broader observational sample in various observational settings.

## Conclusion

Every year, patient boards receive an increasing amount of complaints from patients and significant others regarding healthcare providers’ uncaring behaviour. The CBCS has the potential to reduce these complaints by illuminating healthcare providers’ caring and non‐caring behaviour, as it intertwines the theoretical dimensions of caring behaviour with how to practice caring behaviour. The CBCS can contribute to developing creative interventions in future research, educational curricula, simulation practice and clinical practice with the aim of highlighting how to practice caring behaviour with the intended outcome of patient well‐being.

## Author contributions

Sophie Mårtensson was involved in designing the study, developing and testing the coding schema as well as drafting the manuscript. Eric A. Hodges was involved in designing the study, developing and testing the coding schema as well as drafting the manuscript. Susanne Knutsson was involved in designing the study, developing and testing the coding schema as well as drafting the manuscript. Carina Hjelm was involved in coding the observational data. Anders Broström was involved in developing and testing the coding schema as well as drafting the manuscript. Kristen Swanson was involved in developing the coding schema as well as drafting the manuscript. Maria Björk was involved in designing the study, developing and testing the coding schema as well as drafting the manuscript.

## Ethical approval

This study followed the Helsinki declaration. All students in the course received oral and written information about the study which emphasised confidentiality, that participation was voluntary, and that non‐participation or withdrawal would not affect their grades. After having had opportunity to ask questions, students gave their written informed consent. The study was approved by the Research Ethics Committee in Linköping, Sweden, (DNR 2017/503‐31).

## Funding

This study was supported by School of Health and Welfare, Jönköping University. The authors declare that there is no conflict of interest.
